# Modeling interregional research collaborations in German biotechnology using industry directory data

**DOI:** 10.1016/j.dib.2018.11.145

**Published:** 2018-12-04

**Authors:** Timo Mitze, Falk Strotebeck

**Affiliations:** a Department of Business and Economics, University of Southern Denmark, Denmark; b RWI Essen, Germany; c Rimini Centre for Economic Analysis (RCEA), Canada; d Fachhochschule Südwestfalen, Germany

**Keywords:** Biotechnology, Research collaborations, Industry directory data, Regional innovation system, S&T policy

## Abstract

This article describes a data set to map and model research collaborations in German biotechnology. Underlying micro-data for firms and institutions in the biotech sector together with information on their research collaboration partners have been extracted from a commercial industry directory, the BIOCOM Year and Address book, for 2005 and 2009. The data have been processed and aggregated to the level of NUTS3 regions. This core data set has been linked to regional covariates which measure the regional endowment with biotech-related research capacities, sector-specific S&T policy support and the strength of a region׳s overall local innovation system. The full data set, which is attached to this article, offers applied researchers an alternative source of information for empirical analyses of knowledge flows in research networks and for studying their determinants. Potential fields of application include social network and regression analysis. First empirical results are reported in “Determining factors of interregional research collaboration in Germany׳s biotech network: Capacity, proximity, policy?” (Mitze and Strotebeck, 2018) and “Centrality and get-richer mechanisms in interregional knowledge networks” (Mitze and Strotebeck, 2018).

**Specifications table**

**Table:** 

Subject area	*Economics*
More specific subject area	*Bioeconomy, Networks, Innovation, Economic Policy*
Type of data	*EXCEL and STATA data sets*
How data was acquired	*BIOCOM Year and Address book, German Statistical Office, European Patent Office, ZEW Foundation Panel, BBSR, PROFI*
Data format	*Raw and processed data*
Experimental factors	*Micro data on research collaborations in German biotechnology have been extracted from the BIOCOM Year and Address book for 2005 and 2009 (see*https://biocom.de/angebot/specialist-journals-and-books/?lang=en); *this information has been used to construct a regionally aggregated, dyadic data set for knowledge flows between NUTS3 regions in German biotech research network*
Experimental features	*Core data on knowledge flows in biotechnology have been link with further regional covariates and geo information; variables in the full data set have been processed for the use of social network and regression analysis*
Data source location	*Nomenclature of Territorial Units for Statistics (NUTS) Level 3 regions in Germany*
Data accessibility	*Excel and STATA data sets are included as supplementary materials to this article*
Related research article	*Mitze, T., & Strotebeck, F. (2018). Determining factors of interregional research collaboration in Germany׳s biotech network: Capacity, proximity, policy? Technovation, in press,*doi:10.1016/j.technovation.2018.11.001

**Value of the data**

• Data set presented here is freely available to researchers interested in mapping and modeling knowledge flows in German biotechnology.
• Data set stresses the regional dimension of spatial knowledge exchange and uses small-scale NUTS3 regions as nodes of the biotech research network.
• Data set allows researchers to study the importance of local clusters for network formation and the role played by Science and Technology (S&T) policy therein.
• Data can be employed in social network analysis and regression models; it can be updated and extended using publically available data sources.

## 1 Data

Economic systems are characterized by mutual interdependencies among their actors, and the emergence of network and collaboration structures marks a crucial channel for knowledge exchange and diffusion in modern economies. In particular technology-intensive industries are prone towards the development of networks and alliances with interrelated actors as a means to external knowledge access [3]. The data set presented in this article allows to map and model network structures in Germany׳s biotech research network. While earlier empirical contributions on network formation in technology-intensive industries have mainly used indicators for knowledge flows which are based on patent citations or (collaborative) public funding volumes ([4], [5], [6]), the data at hand extracts and processes information from a commercial industry directory for two sample years (2005 and 2009). The data stresses the spatial dimension of knowledge flows by using small-scale Nomenclature of Territorial Units for Statistics (NUTS) Level 3 regions as nodes of the national biotech research network. Linkages between nodes of the network are measured through research collaborations between actors located in different NUTS3 regions. The full data set, which is which is attached to this article, also contains further regional covariates related to research capacities in biotechnology, the general regional innovation system (RIS) and Science and Technology (S&T) policy support to the regional biotech sector. This allows applied researchers to study the importance of the regional research and knowledge base for network formation in the biotech sector as well as assess the role played by geographical distance, local clusters and S&T policy therein.

## 2 Experimental design, materials and methods

The data construction involves several steps. In a first step, micro data for Germany׳s biotech industry are extracted from the BIOCOM Year and Address book [7], [8]. The BIOCOM AG is a specialized information provider for the European biotech industry and publishes its Year and Address book on an annual basis (see https://biocom.de/angebot/specialist-journals-and-books/?lang=en). The BIOCOM industry directory has become a main source of information for the German biotechnology and, as shown in [1], provides a sufficing coverage of the German biotech sector. The industry directory contains basic information for active biotech firms and institutions, such as their geographical location, foundation year and number of employees, together with details on their active collaboration partners. Data entries in the BIOCOM Year and Address book contain information on registered firms and institutions, which are retrieved from standardized surveys. An exemplary data entry (translated into English) for Apogenix GmbH listed in the BIOCOM Year and Address book 2009 is given below (see [8], p. 53):

**Apogenix GmbH**.

Im Neuenheimer Feld 584.

69120 Heidelberg / Baden-Württemberg.

**Tel.:** +49-6221-58608-0.

**Fax:** +49-6221-58608-10.

**eMail:** contact@apogenix.com.

**Web:** www.apogenix.com.

**Contact:** Dr. Jürgen Gamer (VP Business Development).

**Founded:** 2005.

**Employees:** 28.

**Lab:** L1/S1, L2/S2.

**Biotech Segment:** Health Care/Medicine.

**Fields of Expertise:** Proteins and other Molecules.

**Business Orientation:** R&D, Production.

**Collaborations:** DKFZ, Heidelberg; Celonic GmbH, Jülich; Heidelberg Pharma AG; Probiogen AG; Selexis; Universität Stuttgart.

**Research Focus:** Development of innovative drugs, which exert therapeutical effects by influencing the programmed cell death ♦ Indications: Cancer, Graft-versus-Host Disease.

In a second step, the extracted data on collaboration partners for each listed biotech firm and institution are reprocessed manually in order to check data quality and exclude non-research collaborations such as pure supplier relationships (objects of utility like petri dishes or pipettes) and advisory services (such as consultancies). This step is carried out with the help of Internet search. The cleaning of the underlying micro data results in a core set of biotech actors which conduct research and development (R&D) as their main business activity and have a focus on R&D collaborations. Moreover, in order to account for the interdisciplinary nature of the biotech sector, stated collaboration partners which have not been listed in the initial BIOCOM industry directory are added to the data together with basic information on their geographic location. Due to incomplete information and methodical concerns, foreign collaboration partners of registered biotech actors are excluded from the data (see [1] for further details). The final reprocessed micro data-set contains information on the geographical location and national collaboration activities of 1002 biotech actors in 2005 and 994 in 2009.

As the German biotech landscape is characterized by strong regional clusters of biotech activity, in a third step, the micro data are then aggregated to the level of NUTS3 regions. The following three variables are constructed by summing over the underlying micro data: 1) the number of biotech actors per NUTS3 region, 2) the number of intraregional collaborations between actors within each region *i* and 3) the number of interregional collaboration activities of biotech actors located in two different regions *i* and *j*. An illustrative example for data aggregation to the regional level is provided in [1]. Given the dyadic nature of intra- and interregional collaboration activities among the 439 German NUTS3 regions, the data are stored in a 439 × 439 data matrix with the number of intraregional research activities being recorded on the main diagonal of the matrix, while interregional collaboration activities between two regions are stored as off-diagonal matrix entries. As research collaboration activities are undirected knowledge flows, the matrix is symmetric for two matrix cells (i,j) and (j,i). Excel files containing data matrices for both sample years 2005 and 2009 are included in the supplementary materials of this article.

In a final step, further regional covariates are added to the regionally aggregated data set on research collaborations. This extension enables applied researchers to conduct multivariate analyses, for instance, in order to investigate the regional determinants of network formation within the boundaries of a closed national research network (see, for instance, [1] for the estimation of a gravity-type model of network formation in knowledge networks as well as [2] for a related study analyzing the determinants of a region׳s centrality position in research collaboration networks and its variation over time). Regional covariates have thereby been selected as to represent node properties and the relationship between nodes [9]. They include information on the region׳s research capacities in biotechnology, the geographical proximity between region pairs and the volume of public S&T support to the biotech sector received at the regional level (e.g. the volume of individual and collaborative R&D funding). Moreover, indicators for the state of the overall regional innovation system and local agglomeration forces are included. The full list of variables included in the underlying STATA data set (together with source information) is given in Table 1.

**Table 1:** Variable definitions in data set and source information.

**Mnemonic**	**Variable**	**Description**	**Source**
id	Region	Regional identifier for 439 NUT3 regions	Own definition
year	Year	Time identifier for sample years 2005 and 2009	Own definition
collab	Collaborative linkages	Number of (interregional) research collaborations between region *i* and *j*	[7], [8]
loops	Loops	Number of (intraregional) research collaborations of biotech actors within region *i*	[7], [8]
act	Actors	Number of biotech firms and institutions in region *i*	[7], [8]
dist	Geographical distance	Driving time (in minutes) between centroids of region *i* and *j*	Federal Institute for Research on Building, Urban Affairs & Spatial Development (BBSR)
patent	Biotech patent applications	Weighted number of patent applications in biotechnology in region *i* (OECD definition)	European Patent Office (EPO)
pat_cit	Biotech patent citations	Number of patent citations in biotechnology between region *i* and *j,* see [1] for further details on variable construction	OECD RegPAT
rd_ind	Individual R&D funding	Direct funding of biotechnology-related individual R&D projects by federal government in region *i* (in 1000 €)	Projektförderungs- Informationssystem (PROFI)
rd_collab	Collaborative R&D funding	Direct funding of biotechnology-related collaborative R&D projects by federal government in region *i* (in 1000 €)	Projektförderungs- Informationssystem (PROFI)
brc_winner	Awarded winner region in *BioRegio* contest	Binary dummy variable which takes a value of 1 if region *i* is member of an awarded *BioRegion* in *BioRegio* contest	[11], see supplementary materials for list of regions
brc_part	Participating region in *BioRegio* contest	Binary dummy variable which takes a value of 1 if region *i* is member of an participating *BioRegion* in *BioRegio* contest	[11], see supplementary materials for list of regions
mint_emp	MINT employment	Employees trained in mathematics, informatics, natural sciences and technology [MINT] as share of total employment in region *i* (in %)	Federal Employment Agency
hitec_start	High-tech start-ups	Number of start-ups in high-tech industries relative to MINT employees in region *i* (1=100%)	ZEW Foundation Panel
midtec_start	Medium-tech start-ups	Number of start-ups in medium-tech industries relative to MINT employees in region *i* (1 = 100%)	ZEW Foundation Panel
kis_start	KIS start-ups	Number of start-ups in knowledge-intensive services relative to MINT employees in region *i* (1 = 100%)	ZEW Foundation Panel
open	International openness	Share of foreign turnover in manufacturing sector relative to total turnover in the sector in region *i* (1 = 100%)	German Statistical Office
popdens	Population density	Number of inhabitants per area in region *i* (in square kilometers)	German Statistical Office
spec_manuf	Sectoral specialization manufacturing	Sum of squared deviations in employment shares for NACE3 sectors between region *i* and national average	[12]
spec_bserv	Sectoral specialization business-related services	Sum of squared deviations in employment shares for NACE3 sectors between region *i* and national average	[12]
spec_oserv	Sectoral specialization household-related services	Sum of squared deviations in employment shares for NACE3 sectors between region *i* and national average	[12]
EG_manuf	Ellison-Glaeser index manufacturing	Employment in sectors with high Ellison-Glaeser index (>0.005) relative to total employment in region *i*	[12]
EG_bserv	Ellison-Glaeser index business-related services	Employment in sectors with high Ellison-Glaeser index (>0.005) relative to total employment in region *i*	[12]
EG_oserv	Ellison-Glaeser index household-related services	Employment in sectors with high Ellison-Glaeser index (>0.005) relative to total employment in region *i*	[12]

*Note*: For the case that variables are recorded in the data set for more than one sample year, suffixes are added to the mnemonic in order to identify the specific sample year. For instance, collab05 measures the number of (interregional) research collaborations between region *i* and *j* in 2005, while collab09 captures the values for this variable in 2009.

**Fig. 1:**
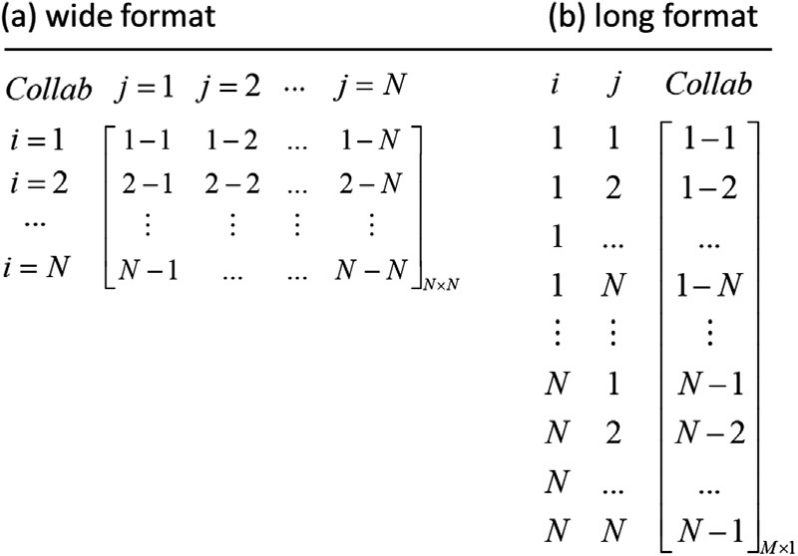
Reorganization of observation on collaboration activities in the STATA data set. *Note*: As outlined in the main text, only data entries on the lower triangular part of the matrix in wide form are stored in the STATA data set (in long format). *Collab* denotes intra- and interregional collaboration activity.

The STATA data set also contains log-transformations of the variables listed in Table 1. Potential users of the data should further note that the data set is organized in dyadic form. With regard to the recorded collaboration activities this implies that the above described *N* × *N* data matrix (with *i*,*j*=1,…,*N* and *N=*439) has been transformed from a ‘wide’ format to a ‘long’ format organized as a (*M*×1) variable vector with *M*=(*N*+1) × *N* observations. The transformation from a ‘wide’ to a ‘long’ format is shown in Fig. 1 below. Further, in order to avoid an over-precision bias due to double counts (given that the collaboration data are undirected), only observations on the lower triangular part of the original *N* × *N* data matrix are used, which results in a total number of (440×439)/2=96,580 observations in the attached STATA data set. While collaboration activities and the geographic distance vary for each (i,j)-region tuple, all remaining regressors are measured at the regional level *i*. As regression analyses typically require that all variables are measured in dyadic form, the STATA data set also includes average values for log-transformed variables such as $x_{\mathrm{ij}}=\left(x_{i}+x_{j}\right)/2$ thereby following the large literature on Gravity models [10]. In the STATA data set, these transformed variables are labelled with the suffix “_ij”, while the original variables are labeled as “_i” and “_j”. Further descriptive information on the data structure are given in [1].

**Fig. 2:**
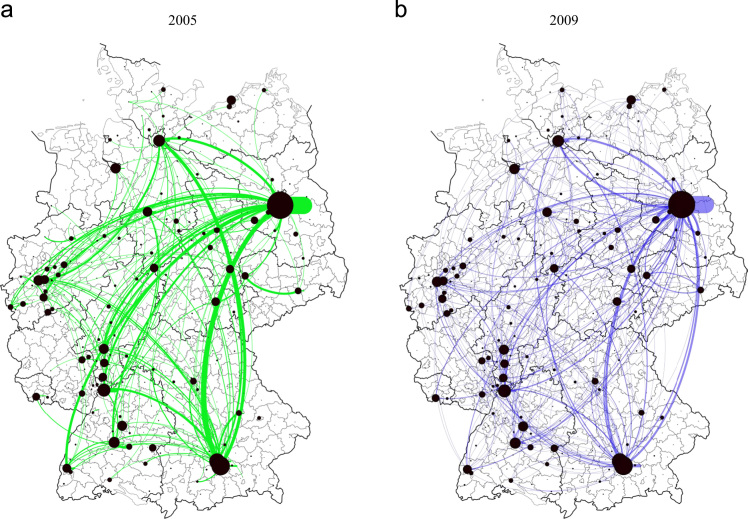
Distribution of intra- and interregional research collaboration activities in biotech network of German NUTS3 regions. *Source:* Own figure based on data from BIOCOM (2005, 2009).

Fig. 2 provides a graphical overview of the regional distribution of intra- and interregional research collaboration activities between German NUTS3 regions in 2005 and 2009. While dots mark the centroids of NUTS3 regions, the dot size indicates the number of active intraregional collaborations. Similarly, while curved lines present active interregional research collaboration activities between two NUTS3 regions, the line width is a measure for the strength of these pairwise interactions. As the figure shows, the national collaboration network in German biotechnology is mainly characterized by knowledge flows within and between distinct urban centers such as Berlin, Munich and Hamburg plus strong macro-regional clusters of biotech activity in the Rhine area (Cologne) and the Rhine-Neckar area (around Heidelberg). In most cases, these macro-regional clusters are organized as so-called *BioRegions*, that is, institutionalized cluster initiatives which have been formed in the mid-1990s as a response to a network- and cluster-oriented public funding regime in the German biotech industry. Details on the evolution of the German biotech industry, the formation of *BioRegions* and the role of public funding therein are provided in the supplementary materials of this article. Further information can also be found in [1], [2] and [13].

**Fig. 3:**
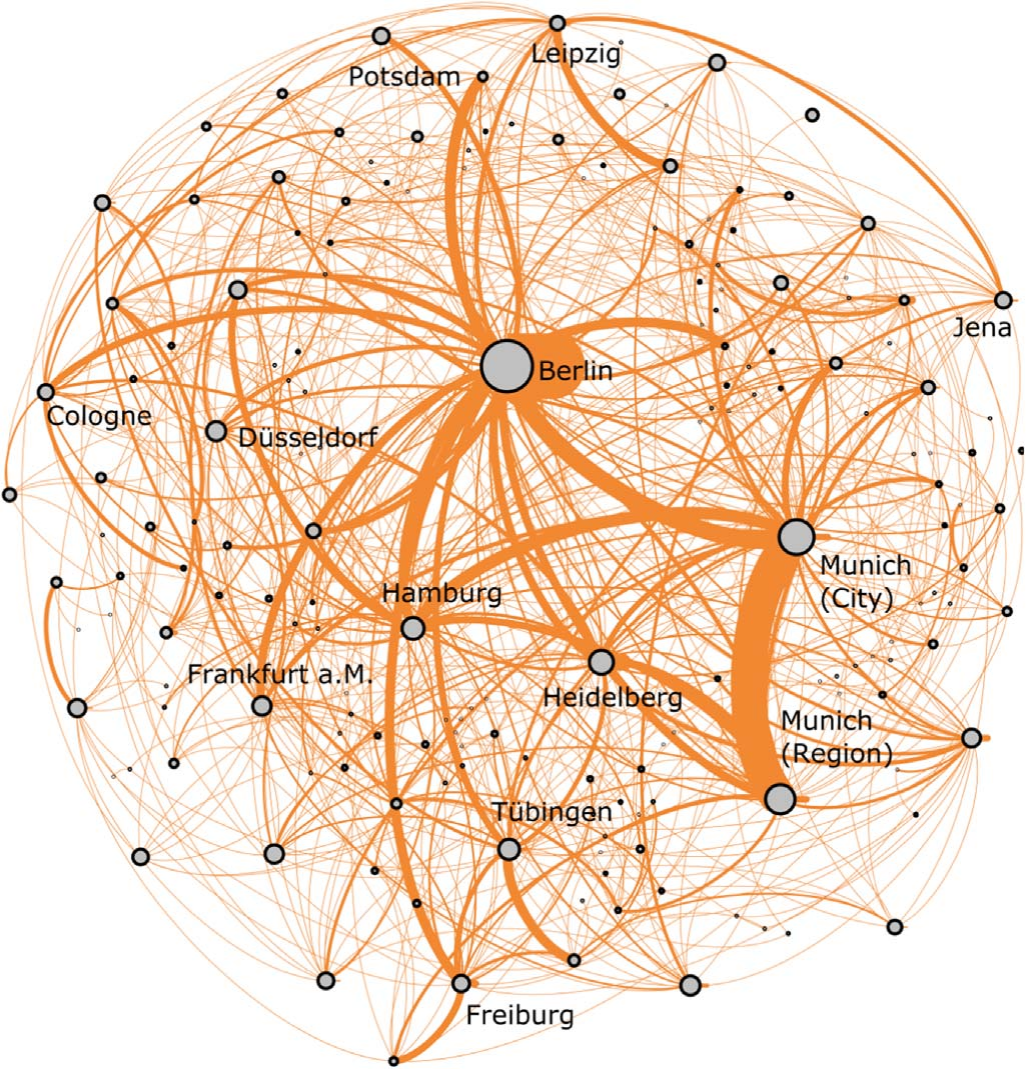
Force-directed graph of German biotech research network in 2005. *Source:* Own figure based on data from BIOCOM (2005).

The presence of clusters with strong interactions between the regional nodes of Germany׳s national biotech network can also be seen when the visualization of the network is reorganized to a force-directed graph as done in Fig. 3 for the year 2005. A force-directed graph organizes node locations in such a way that more strongly related nodes (measured through their linkages) are placed in closer proximity to each other, while unrelated nodes are placed farther apart. Since force-directed graphs additionally organize networks in such a way that all linkages between nodes are of more or less equal length with as few crossing linkages as possible, this implies that nodes with many linkages are placed in the center of the network. Fig. 3 thus clear highlights that urban centres such as Berlin, Munich and Hamburg with strong intra- and interregional collaboration activities are in the centre of the national biotech network. Moreover, several smaller nodes (measured in terms of their intraregional collaboration activity) which are part of a macro-regional *BioRegion* cluster, such as Heidelberg, Freiburg and Jena, are also positioned in close proximity to the center of the network.

As shown in greater detail in [1], the research collaboration network constructed on the basis of data from the BIOCOM Year and Address book is significantly correlated with the spatial pattern of biotech-related patent citations across German NUTS 3 regions. Although the visual inspection of panel a and panel b in Fig. 2 suggests that interregional research collaborations are stable over time, an early analysis [2] has shown that the number and direction of research collaborations (both at the individual and regional levels) have changed significantly between 2005 and 2009. This implies that the data may also be used to analyse the short-term evolution of knowledge networks as done in [2].

Table 2 reports summary statistics for the variables included in the data set. These variables are used in [1] for estimating a gravity-type model of interregional knowledge flows in German biotechnology. Table 3 displays pairwise correlation coefficients for the variables in the data set together with an indicator for their statistical significance. Several statistically significant bivariate correlations can be detected: For instance, interregional collaborations are reported to be strongly correlated over time (0.715), while for both sample years the degree of interregional correlation activity is negatively correlated with the geographical distance between regional nodes (-0.037 and -0.042, respectively). Further, the pattern of interregional patent citations in biotechnology is positively correlated with the extent of interregional research collaborations as measured on the basis of the BIOCOM data. Finally, the pairwise correlation coefficients shown in Table 3 also report several significantly positive correlations between the degree of interregional research collaborations and regional (research) endowments, for instance, related to the amount of research funding in biotechnology, the share of MINT employees in total regional employment as well as the regional start-up activity in knowledge-intensive services and the region׳s overall population density. A more elaborate econometric analysis of the underlying correlations among the variables with focus on the determining factors of network formation is given in [1].

**Table 2:** Sample period and summary statistics of variables.

**Variable**	**Year**	**Mean**	**Std. Dev.**	**Min.**	**Max.**
Collaborative linkages (collab05)	2005	0.011	0.189	0	28
Collaborative linkages (collab09)	2009	0.011	0.200	0	35
Loops (loops05)	2005	0.711	4.175	0	102
Loops (loops09)	2009	0.584	3.473	0	87
Actors (act)	2005	2.066	7.565	0	100
Geographical distance (dist)	2005	308.04	153.27	0	844.50
Biotech patent applications (patents)	Sum of 1997–2002	13.55	47.95	0	576.06
Biotech patent citations (pat_cit)	Sum of 1997–2005	0.006	0.458	0	86
Individual R&D funding (rd_ind)	Sum of 1997–2002	1869.00	10941.57	0	132214.60
Collaborative R&D funding (rd_collab)	Sum of 1997–2002	958.96	4913.41	0	61094.43
BioRegio winner (brc_winner)	Binary dummy	0.023	0.151	0	1
BioRegio participant (brc_part)	Binary dummy	0.050	0.217	0	1
BioRegio interaction dummy1 (brc winner × brc winner)	Binary dummy	0.001	0.035	0	1
BioRegio interaction dummy2 (brc winner × brc part)	Binary dummy	0.004	0.063	0	1
BioRegio interaction dummy3 (brc part × brc_part)	Binary dummy	0.004	0.060	0	1
High-tech start-ups (hitech_start)	Average 1996–2003	0.005	0.003	0.001	0.021
Medium-tech start-ups (midtec_start)	Average 1996–2003	0.007	0.003	0.001	0.036
KIS start-ups (kis_start)	Average 1996–2003	0.061	0.020	0.021	0.154
International openness (open)	Average 1997–2002	23.705	13.005	0.001	96.186
MINT employment (mint_emp)	Average 1997–2002	2.213	1.288	0.450	13.550
Population density (popdens)	Average 1997–2002	439.993	595.233	40.838	3904.828
Sectoral specialization manufacturing (spec_manuf)	1998	684.894	813.730	144.480	8120.970
Sectoral specialization business-related services (spec_bserv)	1998	267.135	199.279	39.130	2911.669
Sectoral specialization household-related services (spec_oserv)	1998	148.847	103.465	19.530	609.070
Ellison-Glaeser index manufacturing (EG_manuf)	1998	26.750	38.052	0.210	390.832
Ellison-Glaeser index business-related services (EG_bserv)	1998	12.243	39.096	0.054	460.573
Ellison-Glaeser index household-related services (EG_oserv)	1998	7.015	24.861	0.013	279.038

*Notes*: See Table 1 for variable definitions.

**Table 3:** Pairwise correlations among variables in data set.

	collab05	collab09	loops05	loops09	Act	dist	patent	pat_cit	rd_ind	rd_collab	brc_winner	brc_w_p	brc_part
collab05	1.000												
collab09	0.715^*^	1.000											
loops05	0.125^*^	0.143^*^	1.000										
loops09	0.120^*^	0.142^*^	0.930^*^	1.000									
act	0.251^*^	0.226^*^	0.550^*^	0.517^*^	1.000								
dist	−0.037^*^	−0.042^*^	−0.021^*^	−0.021^*^	0.023^*^	1.000							
patent	0.090^*^	0.082^*^	0.134^*^	0.129^*^	0.347^*^	−0.051^*^	1.000						
pat_cit	0.024^*^	0.019^*^	0.025^*^	0.027^*^	0.047^*^	−0.015^*^	0.025^*^	1.000					
rd_ind	0.125^*^	0.117^*^	0.224^*^	0.217^*^	0.483^*^	−0.041^*^	0.443^*^	0.026^*^	1.000				
rd_collab	0.123^*^	0.112^*^	0.198^*^	0.195^*^	0.466^*^	−0.005	0.506^*^	0.033^*^	0.651^*^	1.000			
brc_winner	0.065^*^	0.041^*^	0.023^*^	0.015^*^	0.075^*^	−0.032^*^	0.060^*^	0.129^*^	0.060^*^	0.080^*^	1.000		
brc_w_p	0.052^*^	0.037^*^	0.038^*^	0.033^*^	0.104^*^	0.002	0.087^*^	0.013^*^	0.114^*^	0.128^*^	−0.002	1.000	
brc_part	0.025^*^	0.032^*^	0.030^*^	0.042^*^	0.069^*^	−0.013^*^	0.064^*^	0.062^*^	0.113^*^	0.106^*^	−0.002	−0.004	1.000
mint_emp	0.093^*^	0.085^*^	0.151^*^	0.146^*^	0.345^*^	−0.034^*^	0.377^*^	0.024^*^	0.426^*^	0.439^*^	0.072^*^	0.112^*^	0.090^*^
hitec_start	0.015^*^	0.013^*^	0.000	−0.002	0.047^*^	−0.028^*^	0.143^*^	0.003	0.130^*^	0.149^*^	0.025^*^	0.024^*^	0.00^*^4
midtec_start	−0.001	−0.004	−0.018^*^	−0.021^*^	−0.018^*^	−0.048^*^	0.084^*^	−0.005	0.016^*^	0.033^*^	0.000	−0.012^*^	−0.023^*^
kis_start	0.078^*^	0.068^*^	0.086^*^	0.084^*^	0.280^*^	−0.038^*^	0.472^*^	0.018^*^	0.381^*^	0.371^*^	0.048^*^	0.074^*^	0.059^*^
open	0.022^*^	0.015^*^	0.032^*^	0.028^*^	0.075^*^	−0.073^*^	0.208^*^	0.008^*^	0.053^*^	0.099^*^	0.006	−0.002	−0.014^*^
popdens	0.089^*^	0.079^*^	0.152^*^	0.150^*^	0.350^*^	−0.056^*^	0.331^*^	0.027^*^	0.349^*^	0.327^*^	0.071^*^	0.118^*^	0.102^*^
spec_manuf	0.019^*^	0.021^*^	0.041^*^	0.024^*^	0.095^*^	0.015^*^	0.036^*^	0.008^*^	0.062^*^	0.036^*^	0.023^*^	0.041^*^	0.039^*^
spec_bserv	−0.012^*^	−0.005	−0.022^*^	−0.017^*^	−0.040^*^	−0.009^*^	−0.123^*^	0.000	−0.104^*^	−0.071^*^	−0.007^*^	−0.021^*^	−0.029^*^
spec_oserv	0.000	0.008^*^	−0.001	0.023^*^	−0.007^*^	0.019^*^	−0.054^*^	0.008^*^	−0.011^*^	−0.015^*^	0.012^*^	0.001	−0.018^*^
EG_manuf	−0.089^*^	−0.087^*^	−0.181^*^	−0.206^*^	−0.353^*^	−0.066^*^	−0.197^*^	−0.023^*^	−0.309^*^	−0.263^*^	−0.023^*^	−0.041^*^	−0.039^*^
EG_bserv	−0.073^*^	−0.066^*^	−0.137^*^	−0.136^*^	−0.286^*^	0.024^*^	−0.165^*^	−0.013^*^	−0.178^*^	−0.166^*^	−0.002	−0.006	−0.008^*^
EG_oserv	−0.066^*^	−0.060^*^	−0.119^*^	−0.117^*^	−0.257^*^	0.034^*^	−0.181^*^	−0.010^*^	−0.139^*^	−0.139^*^	0.000	0.000	−0.001
	mint_emp	hitec_start	midtec_start	kis_start	open	popdens	spec_manuf	spec_bserv	spec_oserv	EG_manuf	EG_bserv	EG_oserv	

collab05													
collab09													
loops05													
loops09													
act													
dist													
patent													
pat_cit													
rd_ind													
rd_collab													
brc_winner													
brc_part													
mint_emp	1.000												
hitec_start	0.179^*^	1.000											
midtec_start	0.095^*^	0.386^*^	1.000										
kis_start	0.483^*^	0.393^*^	0.252^*^	1.000									
open	0.112^*^	0.134^*^	0.266^*^	0.220^*^	1.000								
popdens	0.590^*^	0.135^*^	0.067^*^	0.431^*^	0.095^*^	1.000							
spec_manuf	0.082^*^	−0.045^*^	−0.152^*^	0.059^*^	−0.099^*^	0.248^*^	1.000						
spec_bserv	−0.053^*^	−0.051^*^	−0.141^*^	−0.097^*^	−0.091^*^	−0.137^*^	0.178^*^	1.000					
spec_oserv	−0.003	−0.013^*^	−0.086^*^	−0.059^*^	−0.043^*^	−0.151^*^	0.088^*^	0.213^*^	1.000				
EG_manuf	−0.262^*^	0.114^*^	0.098^*^	−0.019^*^	−0.016^*^	−0.127^*^	−0.051^*^	−0.205^*^	−0.323^*^	1.000			
EG_bserv	−0.064^*^	−0.052^*^	−0.020^*^	−0.049^*^	−0.070^*^	−0.021^*^	−0.149^*^	−0.259^*^	−0.166^*^	0.570^*^	1.000		
EG_oserv	−0.016^*^	−0.071^*^	−0.048^*^	−0.081^*^	−0.102^*^	−0.002	−0.130^*^	−0.242^*^	−0.142^*^	0.455^*^	0.955^*^	1.000	

*Note*: ^*^ denotes statistical significance at the 5% critical level. All variables are expressed in dyadic form (see main text for further details on variable transformations). See Table 1 for variable definitions.

Although the data are currently organized in a cross-sectional manner, the data set can be easily extended to a panel structure as done in [2] for a subset of variables presented here. Moreover, the BIOCOM industry directory is published on an annual basis, which allows applied researchers to further extend the sample range beyond the two yearly snapshots presented here. It is also possible to update the collaboration data in regular intervals. However, unfortunately the BIOCOM Year and Address book is only available as a print issue so that the underlying micro information used for the construction of this data set need to be digitized manually. Finally, most of the included regional covariates listed in Table 1 are publically available as well.
