# Supplementary material for: Modeling interregional research collaborations in German biotechnology using industry directory data
Source: Data Brief. 2018 Dec 4;22:169–80. doi: 10.1016/j.dib.2018.11.145 (PMC6301979; doi:10.1016/j.dib.2018.11.145)
Supplement: Supplementary file 1 — Supplementary material. [file mmc1.pdf]

CONFLICT OF INTEREST FORM

Manuscript Title: Modeling interregional research collaborations in German biotechnology using industry directory data

Manuscript Number: DIB-18-3052

Journal: Data in Brief

Author(s): Timo Mitze, Falk Strotebeck

The corresponding author confirms on behalf of all authors that there have been no involvements that might raise the question of bias in the work reported or in the conclusions, implications, or opinions stated:

Printed Name (corresponding author): Timo Mitze

Signature: ______________________________

Date: 29.11.2018
