# Supplementary material for: Modeling interregional research collaborations in German biotechnology using industry directory data
Source: Data Brief. 2018 Dec 4;22:169–80. doi: 10.1016/j.dib.2018.11.145 (PMC6301979; doi:10.1016/j.dib.2018.11.145)
Supplement: Supplementary file 4 — Supplementary material. [file mmc4.pdf]

Evolution of German biotechnology in an international context

While the German economy was characterized by having a merely inhospitable climate for biotechnology two decades ago (Dickman, 1996), today the country has developed to one of Europe's leading biotechnology countries. As Müller (2002) outlines, the number of dedicated biotechnology firms has grown from 17 to 333 in the period between the early 1990s and 2001 – thereby outpacing the number of biotech firms in the UK (271) and France (240). More recent data from the European cluster observatory (2016) show that the German biotechnology industry has manifested its leading role as national biotechnology player within Europe since then. Figure A.1 plots different indicators characterizing the national innovation systems in biotechnology with regard to industry concentration (location quotient, see Isserman, 1977), overall industry size as well as a composite "stars" indicator for the period 1995-2010. The European cluster observatory thereby defines the biotechnology industry according to the 4-digit level NACE Rev. 2 classification as 72.11 "Research and experimental development on biotechnology", which is in line with similar studies as in Laskawi (2015).

The location quotient (LQ) in panel (a) of Figure A.1 compares the extent to which the national economies of Germany, the UK, France, and Switzerland have an above average concentration of biotechnology employment relative to the EU-27 average (indicated by LQ ≥ 1). As the figure shows, the industry concentration in Germany and the UK has been constantly above the EU-27 average for the sample period 1995-2010. In comparison, biotechnology-related employment concentration in Switzerland has rapidly grown in the second half of the last decade, while the LQ value for France has dropped considerably. Reasons for the striking decline in French biotechnology can be attributed to an increasing relocations of firms (particularly to Switzerland), a funding crisis with a drastic decline in equity investments and no active financial market as back-up (PharmaLetter, 2005a&b, BioSpace, 2009) combined with the worsening of the overall macroeconomic conditions in the course of the global financial and economic crisis (France Biotech, 2008).

Figure A.1. Evolution of biotechnology industry in selected European countries (1995–2010)

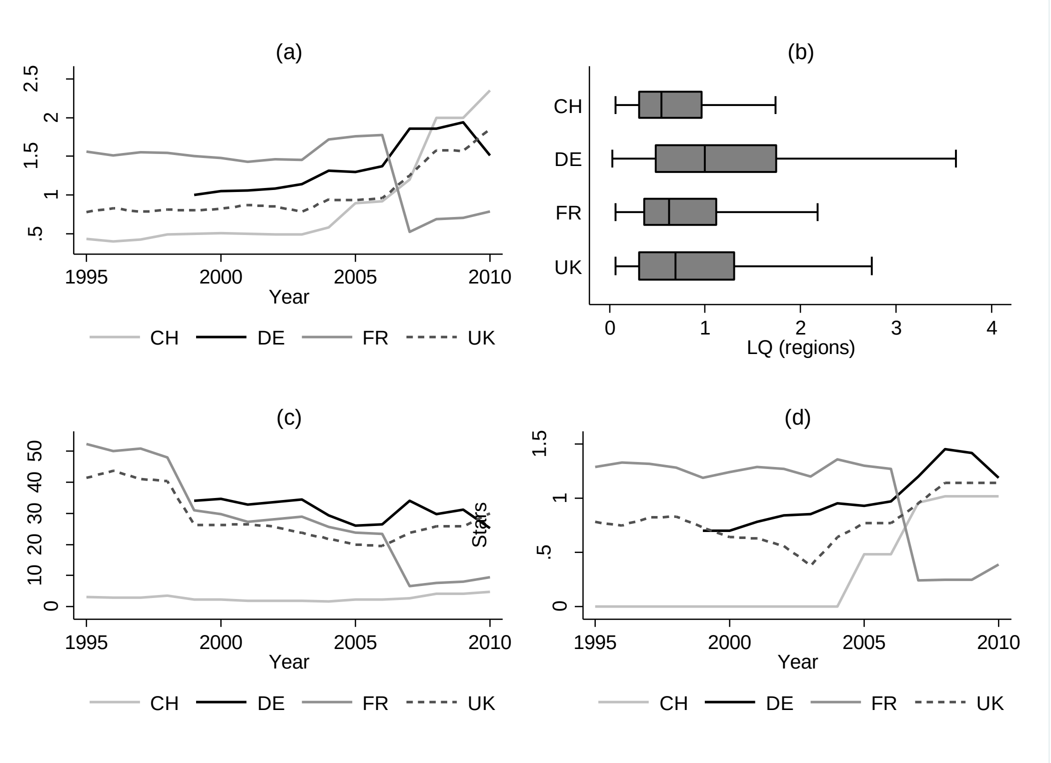

Notes: CH = Switzerland, DE = Germany, FR = France, UK = United Kingdom. The four panels display the following information: (**a**) LQ (country); (**b**) LQ (regions); (**c**) size and (**d**) observatory star rating. For details on the definitions of these indicators see main text; calculated on the basis of data from the European cluster observatory (2016).

In addition to the computation of national LQ values for the biotechnology sector in the four economies, panel (b) in Figure A.1 shows a box plot graph of the intra-national distribution of LQ values in biotechnology at the level of NUTS2 regions (again measured relative to the EU-27 average). As the box plot graph shows, Germany has both the highest median value in terms of regional LQ values as well as particularly the highest LQ values for regions in the upper quartile of the distribution indicating that Germany hosts the most highly concentrated hot spots of European biotechnology employment. Looking at the absolute size of the biotechnology in the four economies (measured in terms of thousands of employees), panel (c) in Figure A.1 shows that biotechnology employment in the UK and Germany is above the employment level in France and Switzerland, particularly in recent

years. Again the time series in panel (c) shows that France experienced a considerable drop in employment levels compared to the UK and Germany.

On the basis of different indicators (LQ, size and focus) the European cluster observatory also computes a 'star' ranking as composite indicator for the significance of the industry in a particular country or region (see Crawley and Pickernell, 2012, for a critical appraisal). The focus sub-indicator, which has not been presented here, thereby measures the extent to which the national economy is focused upon biotechnology employment relative to total national employment. The 'star' ranking in panel (d) of Figure A.1 indicates that Germany has outpaced the other three economies with respect to this composite ranking for biotechnology mainly during the recent sample period after 2000.

Further indicators for the evolution of the sectoral innovation system in German biotechnology point to the same direction: Comparing the distribution of patent applications in the EU-28 and the United States for the period 2004-2012, panel (a) of Figure A.2 shows that both economic blocks have an almost equal overall share (U.S. 52%; EU-28 48%). Within the EU-28, Germany has the largest share of patent applications followed by France and the UK. Looking at the evolution of patent applications over the time period 1977 to 2012, panel (b) of Figure A.2 shows that the number of EPO patent applications per million of inhabitants has grown exponentially during the 1990s in selected European economies and the United States. Thereby the German patent dynamics has even matched up with the level of patent applications per million inhabitants in the United States as leading biotechnology nation in the world. However, at the same time the evolution of patent applications since the second half of the 2000s shows a trend reversal, particularly for Germany and the United States. The figure thus underlines that particularly the 1990s and early 2000s mark a period of rising biotech activity in the United States and Europe.

Figure A.2. Patent shares and evolution of patent applications in biotechnology per million inhabitants

(a) Patent Shares

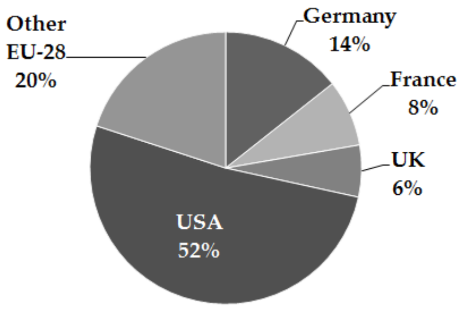

Country	Patents in 2004-2012
Germany	7310.51
France	4026.02
UK	3136.54
Other EU-28	10166.4
USA	26257.89

(b) Patent Evolution

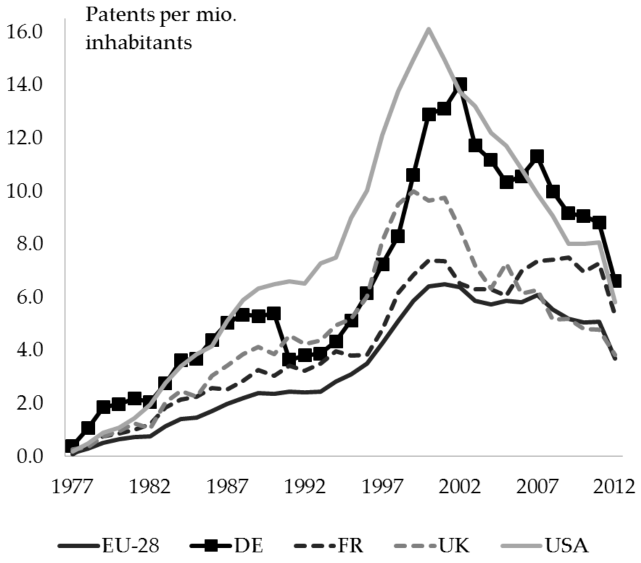

Notes: Panel (a) describes to percentage shares of patent applications for 2004-2012 in the EU-28 and the United States (summed to 100%); panel (b) show the time evolution for selected countries since 1977; both figures calculated on the basis of data from Eurostat (2016).

Taking a closer look at the German development during this period, particularly massive deregulation, public funding, good provision of venture capital, a high rate of innovative start-ups and rapid localized knowledge transfers have been identified the major driving factors for the rapid progress of the industry in the 1990s (see, for instance, Dohse, 2000, Müller, 2002). The rise of the German biotechnology sector was thereby also supported by a global change in the worldwide technological regime of the industry, which evolved from an explorative state-of-art to a merely exploitative one. In the course of this structural shift, codified knowledge (rather than tacit) became increasingly important and facilitated the spreading of new ideas and research collaborations across longer distances (Ter Wal, 2014). These developments paved the way for the emergence of multiple hot-spots of biotech activity in the geographical landscape of the industry's innovation system.

One specific indicator for the change in the technological regime in biotechnology is the growing importance of collaborative R&D activities (see, for instance, Roijakkers and Hagedoorn, 2006, for the specific case of pharmaceutical biotechnology). Public support to collaborative R&D projects thereby

also turned into the main focus of policy makers. As Figure A.3 shows on the basis of data from the PROFI database of the German Federal Ministry for Education and Research (*Bundesministerium für Bildung und Forschung*, BMBF), the share of actors participating in funded collaborative R&D projects (panel (a)) as well as their relative funding volume as part of overall funding in the biotechnology sector (panel (b)) follow a positive long-run growth trend over the period 1982-2007. These growth trends thereby also reflect the paradigmatic shift in the organization of public funding schemes for the biotech industry since the mid-1990s on the basis of competitive funding elements and the promotion of cooperative R&D activities (BMBF, 2005).

Figure A.3. Share of actors and financial volume in publically-funded collaborate R&D projects

(a) Funded Actors *(b) Funding Volume*

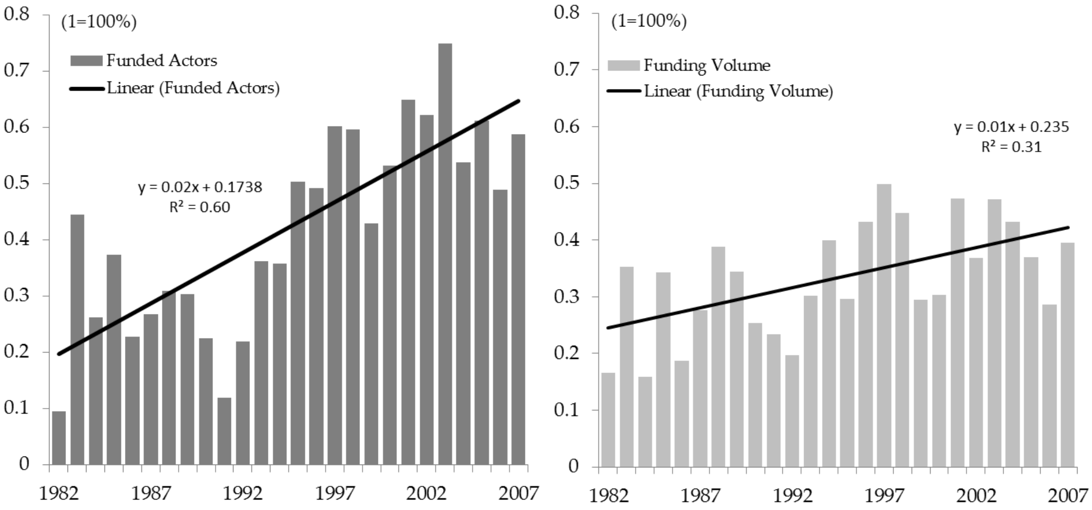

Notes: Panel (a) shows the percentage share of participating actors in collaborative R&D research projects in German biotechnology as part of all funded actors together with a linear trend line for period 1982-2007. Panel (b) shows the share of funding allocated to collaborative R&D research projects in German biotechnology as part of the overall funding volume together with a linear trend line for period 1982-2007. Calculations have been made on the basis of data from the PROFI database of the Federal Ministry of Research and Education. To identify relevant projects, the following technology fields have been selected: i) K - Biotechnology and ii) I19080 - Molecular Bioinformatics.

The *BioRegio* contest, launched in 1995, can thereby be seen as a forerunner for this new type of (cluster) policy schemes, which Eickelpasch and Fritsch (2005) label as 'contests-for-cooperation'. The

main idea of the *BioRegio* contest was to encourage regional cluster initiatives (so-called *BioRegions*) to form a common strategy and apply for subsidies to promote the biotechnology industry in the region (Dohse, 2000). A crucial feature of the *BioRegio* contest was its design as a network and cluster policy since the contest promoted the spatial clustering of biotechnology actors in regional innovation systems (Dohse, 2000). The underlying logic of this policy approach built on predictions from theoretical models in regional science and economic geography, which argued that spatial proximity and clustering of economic activities gives rise to increasing economies of scale through localization and urbanization advantages (McCann, 2013). The funding concept of the *BioRegio* contest thereby aimed at developing a new holistic approach for research and technology policy and was planned to integrate biotechnological capacities and scientific, economic and administrative activities.

Figure A.4. Spatial distribution of observatory star rating in biotechnology for German NUTS2 regions

(a) 1998 *(b) 2009*

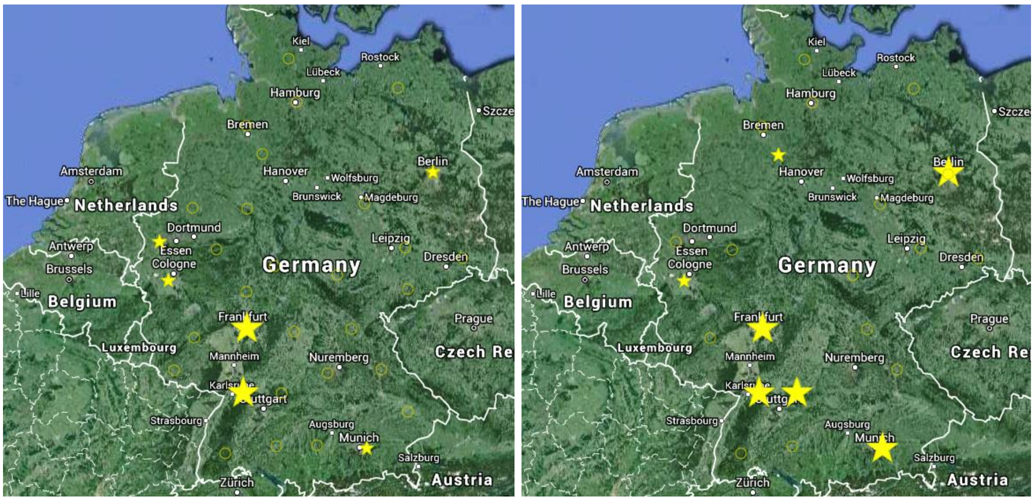

NUTS2	Stars	LQ	Size	Focus	*BioRegion*
Karlsruhe	2	19.37	11.38	1.02	BioRegion Rhein-Neckar-Dreieck
Oberbayern	2	3.2	3.23	0.17	Initiativkreis Biotechnologie München
Berlin	2	2.56	1.66	0.13	BioTOP-Initiative Berlin-Brandenburg
Darmstadt	2	2.46	2.11	0.13	BioRegion Rhein-Main
Stuttgart	2	2.27	2.07	0.12	BioRegion Stuttgart/Neckar-Alb
Köln	1	1.75	1.47	0.09	BioRegion Rheinland
Niedersachsen	1	1.42	2.08	0.07	Region Nordwest-Niedersachsen

Notes: The table shows the values for sub-indicators used to calculate the cluster “stars” in 2009 together with a list of associated *BioRegions*; based on data from the European cluster observatory (2016).

Out of the 17 participating *BioRegions* an independent jury selected four winner regions (Rhineland, Rhine-Neckar, Munich and Jena) of the *BioRegio* contest. Selection criteria were mostly based on "hard" quantitative facts like the existence of a critical mass of biotech firms and research facilities within the region (for details, see Dohse, 2000). As Engel et al. (2013) point out, each winner region received a total lump sum amount of 25 million euro public grants (exception Jena: 15 million euro) for conducting joint R&D projects over a five-year time horizon (1997-2001). Additionally, the winner regions were favored in terms of getting access to the standard public funding schemes of the Federal Ministry of Research and Education. The total amount of these R&D grants exceeded 750 million euros for the time period 1997-2001. With regard to the share of publically funded collaborative R&D projects as outlined in Figure A.3, the four winner regions of the *BioRegio* contest received more than one third of the total collaborative R&D funding provided by the BMBF during the period 1997-2001. The *BioRegio* program was followed by smaller follow-up programmes such as *BioProfile* und *Bio-Chance*.

Besides the purely monetary benefits for awarded *BioRegions*, participating in the contest was also considered attractive for non-winning participants, which could label themselves as part of the national network of *BioRegions* (organized as a registered association, for further information see *Arbeitskreis BioRegionen* https://www.biodeutschland.org/de/ak-bioregio.html) and potentially benefit from signaling effects due to the prestige of the contest. Moreover, the *BioRegio* contest was followed by the *BioProfile* contest starting in 1999 and its winners were mostly selected out of the original pool of *BioRegions*. As Figure A.4 visualizes for the "star" ranking from the European cluster observatory (based on German NUTS2 regions for the sample years 1998 and 2009), these funding programmes led to the prevalence of local clusters and fostered the development of strong regional nodes in the German biotechnology network, which can be linked to the associated *BioRegions* as shown in Figure A.4.

References

BioSpace (2009): France biotech reports a 79% fall in equity investments in French biotech companies in 2008 and asks the government to implement a stimulus plan for young, innovative companies. Available at (last accessed: 05.07.2016): http://www.biospace.com/News/france-biotech-reports-a-79-fall-in-equity/125381.

BMBF (2005): BioRegionen in Deutschland. Starke Impulse für die nationale Technologieentwicklung. 3rd edition, Bundesministerium für Bildung und Forschung (BMBF): Bonn und Berlin.

Crawley, A.; Pickernell, D. (2012): An appraisal of the European Cluster Observatory, in: European Urban and Regional Studies, 19(2): 207-211.

Dickman, S. (1996): Germany joins the biotech race, in: Science, 274: 1454-1455.

Dohse, D. (2000): Technology policy and the regions – the case of the BioRegio contest, in: Research Policy, 29: 1111-1133.

Eickelpasch, A.; Fritsch, M. (2005): Contests for cooperation – A new approach in German innovation policy, in: Research Policy, 34(8): 1269-1282.

Engel, D.; Mitze, T.; Patuelli, R.; Reinkowski, J. (2013): Does cluster policy trigger R&D activity? Evidence from German biotech contests, in: European Planning Studies, 21(11): 1735-1759.

European cluster observatory (2016): Clusters at your fingertips, available at (last accessed 05.07.2016): http://www.clusterobservatory.eu/.

Eurostat (2016): Biotechnology patent applications to the EPO by priority year, available at (last accessed 18.01.2017): http://ec.europa.eu/eurostat/en/web/products-datasets/-/PAT_EP_NBIO

France Biotech (2008): The France biotech annual biotechnology industry report 2007 / 2008. Available at (last accessed 05.07.2016): www.france-biotech.org/en.

Isserman, A. (1977): The location quotient approach to estimating regional economic impacts, in: Journal of the American Institute of Planners, 43(1): 33-41.

Laskawi, C. (2015): Biotechnology. Funding gap jeopardizing competitiveness. Current Issues, Deutsche Bank Research, available at (last accessed 04.07.2016): www.dbresearch.com.

McCann, P. (2013). Modern Urban and Regional Economics. Oxford University Press.

Müller, C. (2002): The evolution of the biotechnology industry in Germany, in: Trends in Biotechnology, 20(7): 287–290.

PharmaLetter (2005a): French biotechnology sector investment is waning, says France Biotech. Available at (last accessed 05.07.2016): http://www.thepharmaletter.com/article/french-biotechnology-sector-investment-is-waning-says-france-biotech.

PharmaLetter (2005b): French biotechnology confronts financial crisis in the industry. Available at (last accessed 05.07.2016): http://www.thepharmaletter.com/article/french-biotechnology-confronts-financial-crisis-in-the-industry.

Roijakkers, N.; Hagedoorn, J. (2006): Inter-firm R&D partnering in pharmaceutical biotechnology since 1975: Trends, patterns, and networks, in: Research Policy, 35(3): 431-446.
