# Supplementary material for: Modeling interregional research collaborations in German biotechnology using industry directory data
Source: Data Brief. 2018 Dec 4;22:169–80. doi: 10.1016/j.dib.2018.11.145 (PMC6301979; doi:10.1016/j.dib.2018.11.145)
Supplement: Supplementary file 5 — Supplementary material. [file mmc5.pdf]

Table B1. List of *BioRegions* in German biotech industry

No.	Name of *BioRegion*	No.	Name of *BioRegion*
**1**	BioTOP-Initiative Berlin-Brandenburg	**10**	BioInitiative Nord
**2**	Region Bremen	**11**	Region Nordwest-Niedersachsen
**3**	BioRegion Freiburg	**12**	BioRegion Regensburg
**4**	BioRegion Greifswald-Rostock	**13**	BioRegion Rheinland
**5**	BioRegion Halle-Leipzig	**14**	BioRegion Rhein-Main
**6**	BioRegion Jena	**15**	BioRegion Rhein-Neckar-Dreieck
**7**	BioMIT Mittelhessen	**16**	BioRegion Stuttgart/Neckar-Alb
**8**	Initiativkreis Biotechnologie München	**17**	Biotechnologie Ulm
**9**	BioRegioN		

Note: Definition of *BioRegions* is taken from Dohse (2007).

Table B2. List of NUTS3 regions as a member of a *BioRegio* winner and participant cluster initiatives

ID	Name of NUTS3 district	*BioRegio* winner	*BioRegio* participant	*BioRegion* number
1002	Kiel	0	1	10
1003	Lübeck	0	1	10
2000	Hamburg	0	1	10
13003	Rostock	0	1	4
13001	Greifswald	0	1	4
3405	Wilhelmshaven	0	1	11
3403	Oldenburg	0	1	11
4011	Bremen	0	1	2
4012	Bremerhaven	0	1	2
3241	Region Hannover	0	1	2
3201	Hannover	0	1	9
3101	Braunschweig	0	1	9
3152	Göttingen	0	1	9
5124	Wuppertal	1	0	13
5111	Düsseldorf	1	0	13
5315	Köln	1	0	13
5313	Aachen	1	0	13
5316	Leverkusen	1	0	13
5354	Aachen	1	0	13
5358	Düren	1	0	13
5314	Bonn	1	0	13
6534	Marburg-Biedenkopf	0	1	7
6531	Gießen	0	1	7
6414	Wiesbaden	0	1	14
6412	Frankfurt	0	1	14
7315	Mainz	0	1	14
6411	Darmstadt	0	1	14
6413	Offenbach	0	1	14

Table B2 (cont'd.). List of NUTS3 regions as a member of a *BioRegio* winner and participant cluster initiatives

ID	Name of NUTS3 district	***BioRegio* winner**	***BioRegio* participant**	***BioRegion* number**
6436	Main-Taunus	0	1	14
6438	Offenbach	0	1	14
7314	Ludwigshafen	1	0	15
7316	Neustadt a. d. W.	1	0	15
8111	Stuttgart	0	1	16
8116	Esslingen	0	1	16
8221	Heidelberg	1	0	15
8222	Mannheim	1	0	15
8416	Tübingen	0	1	16
8415	Reutlingen	0	1	16
8417	Zollernalbkreis	0	1	16
8311	Freiburg	0	1	3
8421	Ulm	0	1	17
9162	München	1	0	8
9188	Starnberg	1	0	8
9362	Regensburg	0	1	12
16053	Jena	1	0	6
15202	Halle	0	1	5
14365	Leipzig	0	1	5
15261	Merseburg-Querfurt	0	1	5
15265	Saalkreis	0	1	5
15154	Bitterfeld	0	1	5
11000	Berlin	0	1	1
12065	Oberhavel	0	1	1
12069	Potsdam-Mittelmark	0	1	1
12072	Teltow-Fläming	0	1	1
12054	Potsdam	0	1	1

Note: The *BioRegion* number refers to the definition given in Table B1.
